# Immunogenic Cell Death and Immunomodulatory Effects of Cabozantinib

**DOI:** 10.3389/fonc.2021.755433

**Published:** 2021-10-20

**Authors:** Fabio Scirocchi, Chiara Napoletano, Angelica Pace, Hassan Rahimi Koshkaki, Alessandra Di Filippo, Ilaria Grazia Zizzari, Marianna Nuti, Aurelia Rughetti

**Affiliations:** Laboratory of Tumor Immunology and Cell Therapy, Department of Experimental Medicine, Sapienza University of Rome, Rome, Italy

**Keywords:** Cabozantinib, immunogenic cell death (ICD), prostate cancer, dendritic cell (DC), TKI (tyrosine kinase inhibitors), extracellular vesicle (EV), HMGB1, immunotherapy

## Abstract

Cabozantinib (XL-184) is a multitarget tyrosine kinase inhibitor (TKI) targeting receptor tyrosine kinases (RTKs) involved in oncogenesis and angiogenesis. It is currently the standard therapy for medullary thyroid cancer (MTC), metastatic renal cell carcinoma (mRCC), and hepatocellular carcinoma (HCC). Combination of Cabozantinib with immunotherapy is now a standard treatment in metastatic renal cancer, and its efficacy is being tested in ongoing clinical trial in prostate cancer patients. Here, we report that Cabozantinib may exert an immunostimulatory role by inducing immunogenic stress of prostate cancer cells and directly modulating dendritic cells (DCs). Cabozantinib treatment arrested the cell cycle and triggered immunogenic cell death (ICD) in prostate cancer cells *in vitro.* Cabozantinib had a direct effect on DCs by the down-modulation of β-catenin and change in migratory and costimulatory phenotype of the DCs. These results may suggest possible immunomodulatory effects induced by Cabozantinib that could be exploited to optimize patient-tailored immunotherapeutic treatments.

## Introduction

Tumor cells and microenvironment cooperate to create an immunosuppressive milieu that promotes tumor development and metastasis and generates a systemic immunosuppressive status, detrimental for the patient. The understanding of these biological-immune mechanisms is crucial to maximize the efficacy of combination therapies for designing patient-tailored approaches (1, 2).

Receptor tyrosine kinases (RTKs) are molecules involved in cell survival, proliferation, motility, and angiogenesis, and their dysregulation promotes cancer transformation (3) and contributes to immunosuppression (4).

Tyrosine kinase inhibitors (TKIs) are small synthetic molecules able to specifically interact and disrupt RTK pathways and are currently adopted in clinical practice for a large number of tumors histotypes (5). Recent findings indicate that besides the direct pharmacological activity against tumor cells, TKI may contribute to antitumor immunity, as it is the case for TKIs targeting the vascular endothelial growth factor receptors (VEGFRs) (6). The VEGF–VEGFR axis is crucial not only for tumor growth and expansion but also for preventing intratumoral immune cell infiltration. Several immune subsets do express the VEGF-Rs and become sensitive to the immunosuppressive action of tumor-released VEGF (7–9). On the other hand, those immune subsets may also become target for TKIs, resulting in a beneficial effect for antitumor immunity (10–12).

Cabozantinib (XL-184) is a multitarget TKI that acts against the VEGF-R2 but also other RTKs (c-MET, RET, KIT, AXL, and FLT3). The Food and Drug Administration (FDA) has approved its use as an anticancer treatment for distinct tumors, such as medullary thyroid cancer (MTC), metastatic renal cell carcinoma (mRCC), and hepatocellular carcinoma (HCC) (13, 14). Recently, Cabozantinib has been approved in combination with immune checkpoint inhibitors (ICIs) as first-line treatment in mRCC (15).

Prostate cancer is the second most common cancer and a leading cause of death in men (16). Due to the pivotal role of androgen receptor in the development of the tumor, androgen deprivation therapy (ADT) is the consolidated treatment in different clinical stages of the diseases (17). However, long-term ADT results in the development of castration-resistant prostate cancer (CRPC), leading to poor prognosis.

Metastatic CRPC (mCRPC) is the most aggressive disease, and the therapeutic options available are Androgen Receptor (AR) signaling inhibitors, docetaxel (cabazitaxel in second-line or postdocetaxel), and radiotherapy, although resistance biological mechanisms occur, leading to death (17).

In prostate cancer, Cabozantinib has been investigated in mCRPC patients as monotherapy in phase II and III clinical trials (18–20) and more recently in combination with docetaxel and prednisone (21).

In on-going active clinical trials, Cabozantinib is administered in combination with ADT (22) and the anti-PD-L1 Atezolizumab (23) in hormone-naive and mCRPC patients, respectively.

In castration-resistant prostate cancer mouse models, Cabozantinib was shown to impact the antitumor immune response by reducing immunosuppression (24) and inducing a strong neutrophil-mediated inflammatory response and accumulation of antigen-presenting cells at the lesion site (25).

Immunogenic cell death (ICD) is a peculiar type of apoptosis characterized by a complex molecular signature: released/newly exposed cell components are recognized as damage-associated molecular patterns (DAMPs) and activate the immune response. Release of the high mobility group box 1 protein (HMGB1), translocation of calreticulin (CRT) from the ER to the plasma membrane, and extracellular adenosine triphosphate (ATP) flux are canonical signs of ICD (26).

ICD is a potent antigenic source for DCs able to cross-present antigen and drive adaptive immune response, activating cytotoxic T lymphocytes (27). In clinical settings, several therapeutic approaches (chemotherapy and radiotherapy) trigger ICD, and this biological potential can synergize with immunotherapy (28, 29).

Here, we investigated the effect of Cabozantinib in the induction of ICD *in vitro* in prostate cancer cells and evaluated its direct effect on monocyte-derived dendritic cells (DCs).

## Material and Methods

### Cell Lines and Chemical Reagents

Prostate cancer DU-145 and PC3 cell lines were purchased from the American Type Culture Collection (ATCC; Washington, DC, NW). DU-145 cells were cultured in Eagle’s minimum essential medium (EMEM) media (ATCC 30-2003) and PC3 cells in F-12K media (ATCC, 30-2004). For each cell line, the medium was supplemented with 10% heat-inactivated fetal calf serum (FCS; Merck KGaA, Darmstadt, Germany), and cells were cultured at 37°C in 5% CO_2_. Cabozantinib (IPSEN, Paris, France) was dissolved in 1N HCl and sterile water (2.5 mg/ml).

### Generation of Dendritic Cells

Human-monocyte-derived DCs were generated from peripheral blood mononuclear cells (PBMCs) of healthy donors (Ethical Committee Protocol, Policlinico Umberto I—“Sapienza” University of Rome, Rif.5282/08.04.2019) as previously described (30). Monocytes (CD14^+^) were purified from PBMCs after Ficoll–Hypaque gradient (1,077 g/ml; Pharmacia LKB) using the Human CD14-Positive Selection Kit (StemCell Technologies, Vancouver, Canada) and cultured (5 × 10^5^ cells/ml) in Roswell Park Memorial Institute (RPMI)-1640 (Sigma-Aldrich) supplemented with 2 mmol/L L-glutamine, penicillin of 100 U/mL, streptomycin of 100 μg/ml (Sigma-Aldrich), with 5% heat-inactivated FCS (Merck KGaA). rhGM-CSF (50 ng/ml) (R&D Systems, Minneapolis, MN, USA) and 2,000 U/ml rhIL4 (R&D Systems) were added at day 0 and 2. Immature DCs (iDCs) were collected on day 5 and matured with cytokine cocktail [rhIL1β, IL6, TNFα (10 ng/ml) all purchased from R&D Systems and PGE_2_ (1 μg/ml) purchased from Sigma-Aldrich)] for 16 h. Cabozantinib (2.5 μg/ml) was added at day 4 during DCs differentiation.

### DC Phenotype

DC phenotype was analyzed by flow cytometry. Briefly, DCs were harvested, washed in phosphate-buffered saline (PBS) and resuspended at 10^6^ cells/ml. Each experimental sample was of 2 × 10^5^ cells/tube (BD Biosciences, NJ, USA), cells were incubated with specific fluorochrome-conjugated antibodies for 30 min at 4°C in the dark. After washing in PBS (two times), the cells were analyzed. The following monoclonal antibodies (mAb) were used: anti-HLAII-DR-APCH7 and anti-CD86-PeCy7, from BD Biosciences; anti-CD14-BB700, anti-CCR7-AlexaFlour647, anti-CD83-Pe, anti-CD40-BB515, anti-ICOS-L-Pe from BioLegend (San Diego, CA); and anti-VEGFR-1-PE from R&D Systems. MoAbs anti-IgG_1_-BB515, -PE, -Pecy7, and -BB700; -AlexaFluor647; and -APC-H7 (BioLegend) were used as isotype controls. All mAbs were purchased from BD Biosciences and BioLegend. Flow cytometry analysis was performed using FACSCanto II flow cytometer running FACS Diva data acquisition and analysis software (BD Biosciences).

### MTT Proliferation Assay

DU-145 and PC3 cells were seeded (2.5 × 10^4^ cell/ml) in 96-well plates (Corning Incorporated, New York, USA) and allowed to adhere overnight. Cells were then treated with serial dilution of Cabozantinib (2.5 and 5 μg/ml) for 24 and 48 h. At the end of each time point, the MTT assay (Roche Diagnostics, Basel, Switzerland) was performed according to the manufacturer’s instructions. Absorbance was measured at 550 nm.

### Assessment of Apoptosis

Cells were seeded in six-well plates (Corning Incorporated; 2.5 × 10^4^ cell/ml) and left to adhere overnight. Cells were treated with serial dilution of Cabozantinib (2.5 and 5 μg/ml) for 24 and 48 h. As control, untreated cells were used. At the end of each time point, cells were harvested by trypsinization (1×, Sigma-Aldrich), resuspended at 10^6^ cells/ml in 1× Annexin V Binding Buffer (BD Biosciences). Cells were stained with 7-AAD and Annexin V-FITC (BD Biosciences) for 15 min to assess cell death. Flow cytometry was performed using FACSCanto II flow cytometer running FACS Diva data acquisition and analysis software (BD Biosciences).

### Cell Cycle Assay

Cells were seeded in a six-well plate (Corning Incorporated; 2.5 × 10^4^ cell/ml) and allowed to adapt overnight. Cells were then treated with serial dilution of Cabozantinib (2.5 and 5 μg/ml) for 24 and 48 h. The cells were fixed in 70% cold ethanol and incubated at 4°C overnight. The cells were incubated with RNaseA (Sigma-Aldrich) for 30 min and stained with propidium iodide (PI) (BD Pharmingen, San Diego, CA, USA). Flow cytometry was performed using FACSCanto II flow cytometer running FACS Diva data acquisition and analysis software (BD Biosciences).

### Cell Lysate and Cytosolic and Nuclear Extracts

iDCs, mDCs, DU-145, and PC3 cells were lysed using radioimmunoprecipitation assay (RIPA) buffer (1×, 100 μl/1 × 10^6^ cells, Cell Signaling, Beverly, MA, USA) supplemented with protease inhibitors (1×, Sigma-Aldrich) for 30 min on ice and then centrifuged at 13,000 *g* for 10 min.

The nuclear and cytoplasmic extracts were obtained from cells with and without serial dilution of Cabozantinib (2.5 and 5 μg/ml) at different time points (24 and 48 h). The cells were resuspended in Buffer A [20 mM HEPES, pH 7.9, 20 mM KCl, 3.0 mM MgCl_2_, 0.3 mM Na_3_VO_4_, and freshly added 200 μM leupeptin, 10 mM E64, 300 μM phenylmethylsulfonyl fluoride (PMSF), 0.5 μg/ml pepstatin, 5 mM dithiothreitol (DTT), and 0.1% Nonidet P-40] and vortexed. After 30 min on ice, cells were centrifuged for 10 min at 10,000 *g* at 4°C. The supernatants were centrifuged another time for 30 min (10,000 *g* at 4°C), and the supernatants were taken as cytosolic extracts and frozen. To obtain nuclear extracts, pellets were resuspended in buffer B [40 mM HEPES, pH 7.9, 0.84 M NaCl, 0.4 mM ethylenediaminetetraacetic acid (EDTA), 50% glycerol, 0.3 mM Na_3_VO_4_, and freshly added 200 μM leupeptin, 10 μM E64, 300 μM PMSF, 0.5 μg/ml pepstatin, and 5 mM DTT] and vortexed. After 1 h on ice, nuclear extracts were cleared at 10,000 *g* for 1 h, at 4°C, and supernatants were transferred to fresh vials. Protein content was quantified by Bradford assay using bovine serum albumin (BSA) as a standard (Bio-Rad Lab, CA, USA), and samples were aliquoted and stored at −80°C.

### Western Blot

Equal amounts of cell lysates or extracts, resuspended in sample buffer (Thermo Fisher Scientific, CA, USA) were resolved using 4%–12% sodium dodecyl sulfate–polyacrylamide gel electrophoresis (SDS-PAGE) gel (Thermo Fisher Scientific) and transferred to nitrocellulose membrane. After blocking with Tris-buffered saline with Tween 20 (T-BST) 5% milk (SERVA Electrophoresis Gmbh, Heidelberg, Germany), membranes were incubated with the following antibodies at a concentration 1:1,000: rabbit anti-β-catenin, anti-AXL, anti-Met, anti-β-tubulin, anti-HSP70, anti-Beclin1, and anti-mTORC all from Cell Signaling; rabbit anti-HMGB1, anti-Calreticulin, and anti-p62 were purchased from Abcam, Cambridge, UK; and anti-β-actin (Sigma-Aldrich, 1:10,000) and mouse anti-LC3 (MBL, Woburn, MA, USA). Membranes were incubated by peroxidase-conjugated goat antirabbit immunoglobulin G (IgG) (H+L; Jackson ImmunoResearch Laboratories; 1:20,000); peroxidase-conjugated goat antimouse IgG (H+L; Jackson ImmunoResearch Laboratories, West Grove, PA USA; 1:20,000). Protein bands were detected with horseradish peroxidase (HRP)-enhanced chemiluminescence (ECL) (Advansta, CA, USA), following the manufacturer’s instructions. The density of protein bands was analyzed by Image J software and was normalized in terms of the average intensity of bands of each protein per the average intensity of bands of β-tubulin or β-actin.

### Calreticulin Membrane Exposure, HMGB1, and ATP Release Evaluation

PC3 and DU-145 cells were seeded in 24-well plates and allowed to adapt overnight. The cells were treated with serial dilution of Cabozantinib (2.5 and 5 μg/ml) for 24 and/or 48 h. Untreated cells were used as experimental control. Calreticulin (CRT) membrane exposure was evaluated by flow cytometry, using as primary antibody anti-calreticulin mouse antibody (Abcam, 1:100), washing cells (two times), and then incubating the cells with PE-conjugated antimouse IgG (Southern Biotech Limited, USA). Flow cytometry was performed using FACSCanto II flow cytometer running FACS Diva data acquisition and analysis software (BD Biosciences). ATP release in the supernatant of cells was measured by means of an ENLITEN ATP Assay kit (Promega, Fitchburg, WI, USA), based on the ATP-dependent luciferin conversion, which yields detectable bioluminescence, according to the manufacturer’s protocol. HMGB1 concentrations in the supernatant of cells were measured by means of an enzyme-linked immunosorbent assay (ELISA) kit (TECAN, Zürich, Switzerland), according to the manufacturer’s protocol.

### Extracellular Vesicles Isolation

Extracellular vesicles (EVs) were purified from the cell culture supernatant of DU-145 cells. To isolate EVs, cells were cultured 3 × 10^5^ cells/ml in EMEM (ATCC) complemented with 2% fetal bovine serum (FBS) (Euroclone) for 24 or 48 h, with or without Cabozantinib (2.5 and 5 μg/ml). The supernatant underwent serial centrifugation steps at 4°C (550 g for 30 min, 1,500 *g* for 30 min). The supernatants were transferred in a fresh tube and then ultracentrifuged at 100,000 *g* for 1 h, at 4°C (Type 35 rotor, Beckman Coulter, USA). After the last ultracentrifugation step, the supernatants were discarded, and the final pellet containing EVs was gently resuspended in PBS without Mg^++^ and Ca^++^ (100 μl/pellet; Sigma-Aldrich), aliquoted and stored at −80°C. Protein concentration was measured by Bradford assay (Bio-Rad Laboratories). Analysis indicated that, in the presence of Cabozantinib, the optimal time point to obtain adequate EV preparation was at 48 h.

### Cellular Uptake and Endocytosis Assays

The uptake of DU-145 released antigens by DCs was first investigated. Briefly, DU145 cells were labeled with carboxyfluorescein succinimidyl ester (CFSE) (1 μM; Life Technologies Thermo Fisher, CA, USA) and incubated for 20 min at 37°C protecting from light. After two washes in PBS (37°C), the cells were treated with 2.5 μg/ml of Cabozantinib (48 h). Cell culture supernatants were collected and added to monocyte-derived DCs at the immature stage for 48 h. At the end of the incubation, the iDCs samples were acquired by a FACSCanto II flow cytometer (Becton Dickinson) and analyzed by FACSDiva software (Becton Dickinson) to verify the transfer of CFSE to the cytoplasm of the cells.

To study the endocytic capacity of DCs cultured in the presence or in the absence of 2.5 μg/ml Cabozantinib, FITC-dextran (1 mg/ml; Molecular Probes, Eugene, USA) was added to DCs for 1 h at 37°C and 4°C. After washing, cells were acquired by FACSCanto II flow cytometer (Becton Dickinson) and analyzed by FACSDiva software (Becton Dickinson).

### Statistical Analysis

Statistical analysis was performed using GraphPad Prism version 8 (Graphpad Software, Inc., San Diego, USA). Descriptive statistics [average and standard error media (SEM)] was used to describe the various data. Student’s paired *t*-test was used to compare two groups. Fold change represents the ratio between values obtained at treated and not treated cells (T/NT). Statistical significance was indicated when the *p*-value was <0.05.

## Results

### Cabozantinib Reduces Tumor Cell Growth by Arresting Cell Cycle in the G1 Phase

The expression of the tyrosine kinase receptors (TKRs) targeted by Cabozantinib was investigated in the DU-145 and PC-3 prostate cancer cell lines. While the normal prostate glandular epithelium expresses low protein levels of AXL and cMET, the cell lines employed in the study displayed distinct profile of TKRs as detected by Western blot and/or flow cytometry and confirmed by public protein expression database (https://www.ebi.ac.uk/gxa/home) (**Supplementary Table S1**). MET and AXL were expressed by both cell lines; in particular, AXL expression was more pronounced in DU-145 cells. Low expression levels of KIT and VEGFR were detected in DU-145 and PC-3 cells, respectively.

**Figure 1:**
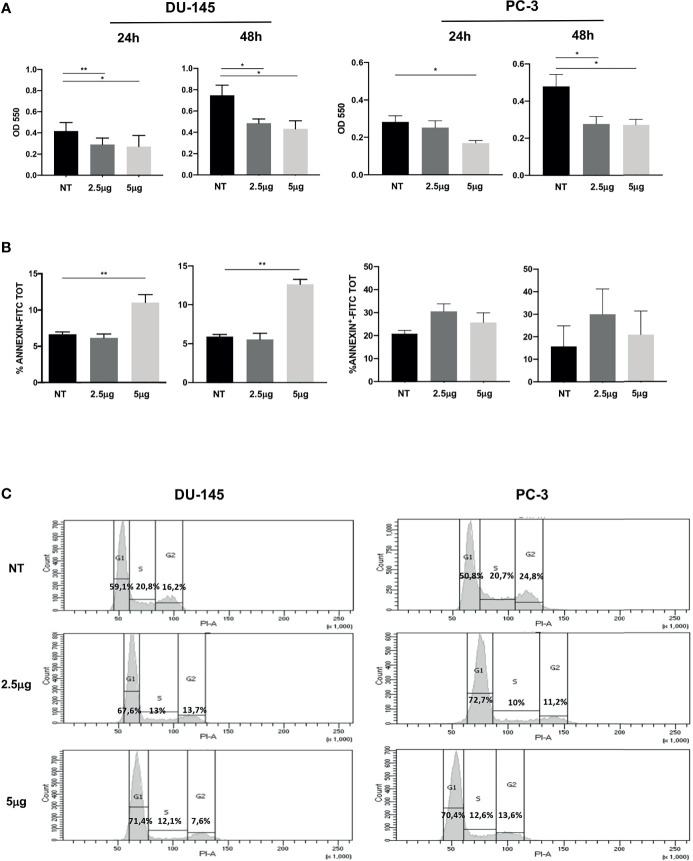
Effect of Cabozantinib on proliferation of DU-145 and PC-3 prostate tumor cells. **(A)** MTT proliferation assay of cells treated for 24 or 48 h with 2.5 and 5 μg/ml of Cabozantinib. Histograms represent the mean values of optical density (OD) 550 nm of three independent experiments (triplicate each condition) ± SEM. **(B)** Apoptosis of cells treated for 24 or 48 h with Cabozantinib (2.5 and 5 μg/ml). Histograms represent the mean values of percentage of apoptotic cells identified as Annexin positive of three independent experiments ± SEM. **(C)** Cell cycle analysis by propidium iodide (PI) staining analyzed by flow cytometry. One representative analysis out of three experiments is shown. The histograms represent the count of PI-positive cells found in untreated (NT) and Cabozantinib-treated (2.5 and 5 μg/ml) DU-145 and PC-3 cells as analyzed by FACS DIVA software. Gates allow the identification of phase G1, S, and G2 cell cycle. NT, untreated cells. *p < 0.05; **p < 0.01, Student’s *t*-test.

To test the impact of Cabozantinib on cell proliferation, cancer cells were cultured in the presence of increasing dose of the drug [2.5 μg/ml, as steady-state plasma concentration achievable in humans (31–33) and 5 μg/ml as exceeding dose], and results are shown in **Figure 1A**. Untreated (NT) cells were used as control.

Cabozantinib induced significant proliferative decrease in DU-145 cells at both drug concentrations and time points tested (at 24 h: *p* < 0.01 NT *vs*. 2.5 μg/ml and *p* < 0.05 NT *vs*. 5 μg/ml; at 48 h: *p* < 0.05 NT *vs*. 2.5 and 5 μg/ml). The response of PC-3 cells to Cabozantinib displayed a different kinetics: at 24 h, a significant proliferative decrease was observed only at the high dose of the drug (5 μg/ml). After 48 h, PC-3 proliferation significantly decreased at both drug concentrations (*p* < 0.01 NT *vs*. 2.5 and 5 μg/ml).

Induction of apoptosis did not appear to make account for the reduced cell growth observed upon Cabozantinib treatment. Indeed, no significant cell death induction was observed in PC-3 cells, independently by the treatment, and in DU-145 cells treated with 2.5 μg/ml of Cabozantinib. Apoptosis was only observed in DU-145 cells at the high drug dose, at both time points (*p* < 0.01) (**Figure 1B**).

These results prompted us to investigate whether Cabozantinib could restrain cell proliferation by cell cycle perturbation. As shown in **Figure 1C**, Cabozantinib treatment triggered cell cycle arrest in G1 phase, in both cell lines. The G1 phase arrest was pronounced at both Cabozantinib concentrations in both cell lines (*p* < 0.01) in the first 24 h, and this effect was maintained in DU-145 at 48 h (**Supplementary Figure S1A**). Concurrently, a decrease in S and G2 phases was observed at different extent in both the prostate cancer cell lines (**Supplementary Figure S1A**).

### Cabozantinib Triggers Cellular Stress and Immunogenic Cell Death

The unbalancing of the cell cycle may result in a strong cellular stress. HMGB1 is a nuclear protein: its cytoplasmic translocation is associated with cellular stress, and its release in the extracellular space indicates severe cell damage.

**Figure 2:**
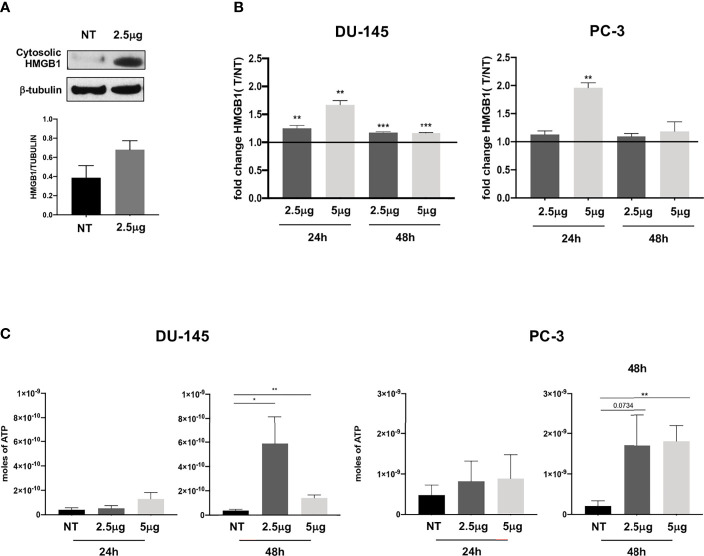
Extracellular release of HMGB1 and ATP following Cabozantinib treatment of DU-145 and PC-3 prostate cancer cell lines. **(A)** Western blot analysis of HMGB1 expression in cytosolic membrane fraction of untreated (NT) or Cabozantinib-treated (2.5 μg/ml) DU-145 cells. β-Tubulin was employed as internal reference standard. Bands intensity was measured by ImageJ software; histograms represent the ratio between HMGB1 (25–29 kDa) and β-tubulin (55 kDa) band intensities. **(B)** HMGB1 release upon Cabozantinib treatment. Histograms represent the ratio between the median values of HMGB1 released by treated cells (2.5 and 5 μg/ml) *vs*. values of HMGB1 released by untreated cells (NT). **(C)** Release of ATP induced by Cabozantinib. The ATP release was measured by ENLITEN-Promega KIT as luminescence signals. The histograms represent the mean value of ATP moles of two independent experiments in duplicate ± SEM. The line on histograms represents the NT (not treated) value. *p < 0.05; **p < 0.01; ***p < 0.001, Student’s *t*-test. NT, untreated cells as experimental control.

Therefore, we evaluated whether Cabozantinib could trigger immunogenic cell death (ICD) of DU-145 and PC-3 defined as HMGB1 release associated with simultaneous release of ATP and calreticulin (CRT) surface exposure. Following Cabozantinib treatment, HMGB1 increased in the cytoplasm fraction of DU-145 cells as detected by WB analysis (**Figure 2A**), and its extracellular release was detected in both cell lines by ELISA assay (**Figure 2B**).

This event was particularly enhanced in the DU-145 cell line at both Cabozantinib concentrations (24 h, *p* < 0.01; 48 h, *p* < 0.001). In the PC-3 cell line, significant HMGB1 release was observed at 24 h of treatment with the higher dose of 5 μg/ml (*p* < 0.01; **Figure 2B**).

**Figure 3:**
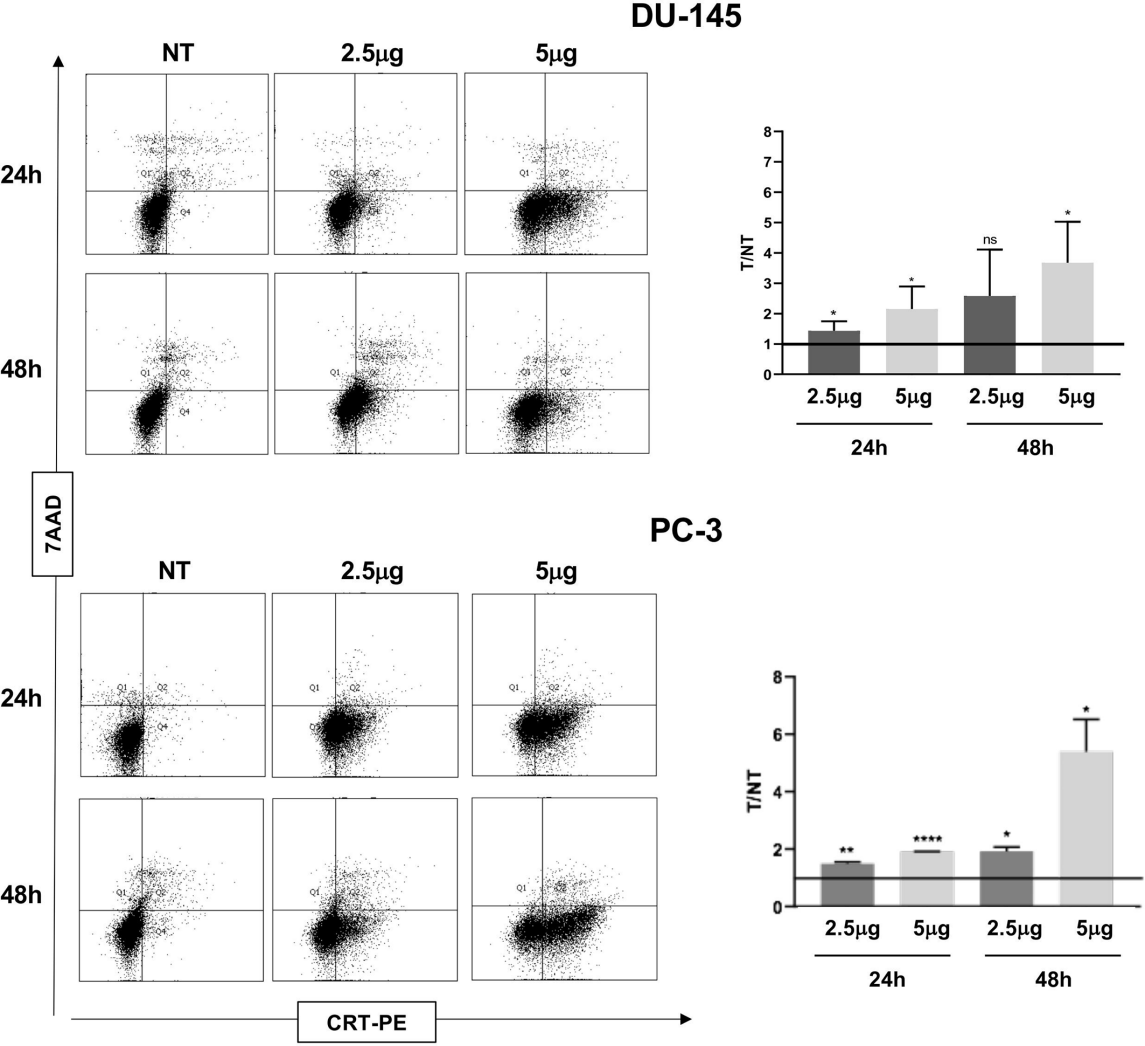
Flow cytometry analysis to evaluate Calreticulin (CRT) cell membrane expression in DU-145 and PC-3 prostate cancer cells treated with Cabozantinib (2.5 and 5 μg/ml) or 24 and 48 h. Dot plots show the analysis of cell population considering the 7-ADD vs. CRT staining (*y- vs. x*-axis, respectively). Histograms represent the ratio (T/NT) between the average of percentage of CRT^+^ cells in Cabozantinib-treated cells (2.5 and 5 μg/ml) *vs*. untreated cells ± SEM, evaluated in three independent experiments. *p < 0.05; **p < 0.01; ****p < 0.0001, Student’s *t*-test. NT, untreated cells as experimental control. ns, not significant.

ATP release, a second feature of ICD, was also significantly induced by Cabozantinib in both cell lines at 48 h (DU-145: *p* < 0.05 NT *vs*. 2.5 μg/ml, *p* < 0.01 NT *vs*. 5 μg/ml; PC-3: *p* < 0.01 NT *vs*. 5 μg/ml) (**Figure 2C**). Then, the CRT exposure was evaluated: both cell lines did express baseline levels of CRT as often observed in cancer cells (**Figure 3**). However, CRT exposure on cell membrane significantly increased in association with Cabozantinib concentration and treatment period (DU-145: *p* < 0.05 at 24 h for both 2.5 and 5μg/ml; *p* < 0.01 NT *vs*. 5 μg/ml at 48 h; PC-3: *p* < 0.01 NT *vs*. 2.5 μg/ml, *p* < 0.0001 *vs*. 5 μg/ml at 24 h; *p* < 0.05 NT *vs*. 2.5 and 5 μg/ml at 48 h).

The detection of the combined ICD markers following Cabozantinib exposure strongly suggests that the drug, although not inducing a cytotoxic effect on prostate cancer cells, impacted the quality of the apoptosis due to the release of ICD markers, with a marked effect on DU-145 cells.

Interestingly, DU-145 cells express high levels of AXL, which is targeted by this TKI. AXL has been shown to be crucial for modulating the balance between ICD and autophagy: AXL overexpression triggers macro-autophagic flux, while its inhibition arrests autophagy, inducing ICD (34). Indeed, Cabozantinib treatment induced upregulation of mTOR in DU-145 cells (negative at baseline) and decreased in the autophagic markers LC3-II and Beclin-1 (**Supplementary Figure S1B**, *p* < 0.05).

These results suggest that Cabozantinib triggers immunogenic cell death and may offer a source of immunogenic tumor antigens.

### Extracellular Vesicles Convey ICD Molecular Signals

The release of EVs mediates the crosstalk among cells in the tissue microenvironment and distant body districts (35). To verify whether tumor EVs contributed to the release of ICD molecular signals, EVs were isolated by ultracentrifugation from the culture supernatant of DU-145 cells grown without or with Cabozantinib (2.5 and 5 μg/ml) for 48 h, which resulted the optimal time point to obtain adequate EV preparation from cells grown in the presence of Cabozantinib.

EVs were analyzed by WB for the expression of ICD-associated molecules HMGB1 and CRT and the stress-associated protein HSP70, which was constitutively expressed in DU-145 cells, independently by TKI treatment (**Supplementary Figure S1C**).

**Figure 4:**
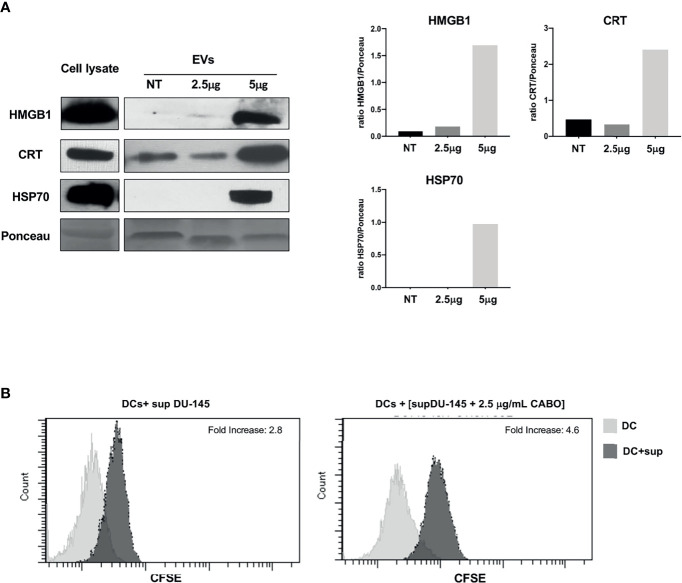
The ICD markers HMGB1, CRT, and HSP70 are released as cargo of EVs shed by Cabozantinib-treated DU-145 cells. **(A)** Western Blot analysis of EV protein cargo (8 μg/sample) obtained from untreated (NT) or Cabozantinib-treated DU-145 (2.5 and 5 μg/ml for 48 h, respectively, 2.5CB and 5CB). As internal control, DU-145 total cell lysates (30 μg/sample) were used. Loading control was performed by Ponceau staining. The histograms represent densiometric evaluation reporting the ratio of band intensity of the sample lane *vs*. Ponceau lane. **(B)** Uptake by DCs of antigens released by untreated and Cabozantinib-treated DU-145. DU-145 cells (treated or untreated) were labeled with CSFE, and DCs were incubated with cell culture supernatants. Transfer of CSFE^+^ antigen to DCs was evaluated by flow cytometry. The negative control is the autofluorescence of DC exposed to DU-145 culture supernatant (gray histograms); the dark histograms represent the fluorescence signals derived from DCs exposed to culture supernatant of CSFE labelled DU-145. Fold increase values are the ratio between the mean fluorescence intensity (MIF) of DCs exposed to CSFE^+^DU-145 cell supernatant and DCs control.

HMGB1, CRT, and HSP70 were detected in the EVs shed by DU-145 cells upon Cabozantinib incubation. CRT appeared to be released associated with EVs also in the untreated cells and increased after treatment (**Figure 4A**); indeed, a baseline expression of CRT was detected in DU-145 cells (**Figure 3**). These results suggest that the damage-associated molecules are released by prostate cancer cells in the microenvironment also by EVs.

To verify whether Cabozantinib-treated tumor cells could be a source of tumor antigens for antigen-presenting cells as such as dendritic cells (DCs), the DU-145 prostate cancer cells were labeled by CFSE and grown in the absence or presence of 2.5 μg/ml Cabozantinib (48 h). The cell culture supernatants were collected and added to monocyte-derived DCs at the immature stage (iDCs) for 48 h. At the end of the incubation, CSFE uptake by iDCs was analyzed by flow cytometry to verify the transfer of CFSE-labeled tumor antigens to DCs (**Figure 4B**). The percentage of CFSE^+^DCs increased after incubation with supernatants derived from CFSE-labeled tumor cells. This percentage further increased when tumor cells were treated with Cabozantinib. These results suggest that tumor cells were able to transfer antigens to DCs probably by the release of EVs and that this uptake increased after the addition of Cabozantinib to tumor cells.

### Cabozantinib Modulates DC Phenotype

We have shown in previous work that DCs biology is modulated by TKI as such as Pazopanib altering the Wnt/β-catenin pathway (10). Indeed, DCs do express VEGFR during their differentiation and may express MET after maturation (data not shown). The addition of Cabozantinib in DC culture during differentiation did not affect their viability (data not shown). We evaluated whether Cabozantinib could affect DCs analyzing the β-catenin signaling and phenotype changes, employing the drug concentration of 2.5 μg/ml.

**Figure 5:**
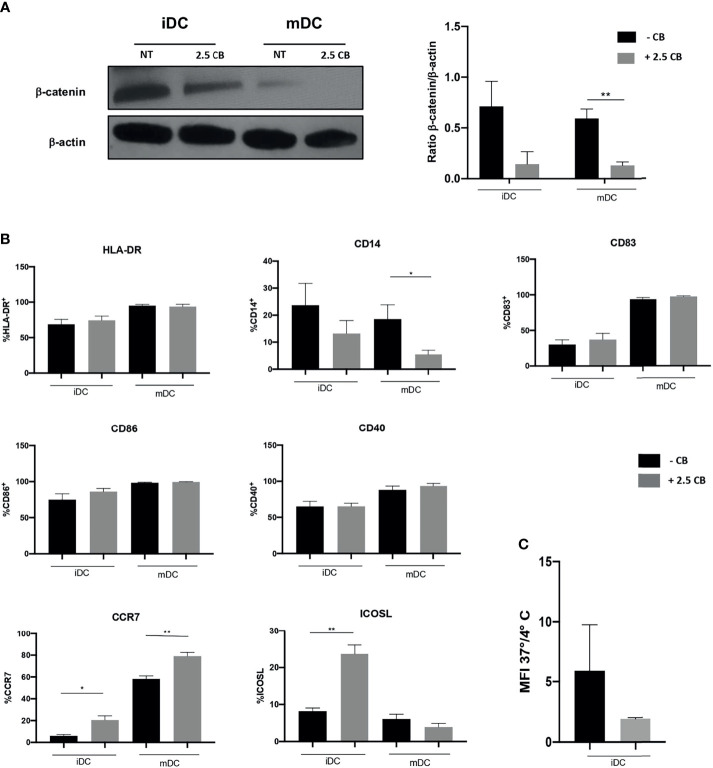
Effect of Cabozantinib on DCs. **(A)** Modulation of β-catenin in DCs upon Cabozantinib treatment. Western blot analysis in iDCs and mDCs, untreated (NT) or treated with 2.5 μg/ml of Cabozantinib (2.5 CB) to detect β-catenin protein (92 kDa). β-Actin (43kDa) was employed as internal reference standard. Band intensity was measured by ImageJ software. Histograms represent normalization of the intensity values obtained as ratio between the sample value and the control. **(B)** The histograms represent the average of MFI values of DC phenotypic markers from healthy donor. The monocytes after 4 days of differentiation were treated with Cabozantinib (2.5 μg/ml), and at day 5, iDCs were collected and matured with cytokine cocktail (rhIL1β, IL6, TNFα, and PGE_2_). The concentration of Cabozantinib used for the culture corresponds to serum levels achieved in TKI-treated patients. The histograms correspond to the average of MFI values among six healthy donors ± SEM. **(C)** Uptake of the fluorescein isothiocyanate (FITC)-dextran by iDCs cultured in presence or absence of Cabozantinib. Results are reported as ratio between the mean fluorescence intensity (MFI) obtained incubating the cells with FITC-dextran for 1 h at 37°C and 4°C. *p < 0.05; **p < 0.01, Student’s *t*-test.

The Wnt/β-catenin pathway stirs the activatory or tolerogenic capacity of DCs: the dampening of this pathway correlates with activation of DCs. Results demonstrated that Cabozantinib reduced the β-catenin pathway in iDCs. This reduction becomes statistically significant after maturation, suggesting the generation of activating mDCs (*p* < 0.01; **Figure 5A**). This metabolic reprogramming corresponded to a phenotype modulation of DCs (**Figure 5B**). Among the DC marker analyzed, the expression of CD14, ICOSL, and CCR7 was significantly modulated by Cabozantinib. In particular, the CD14 molecule, expressed by monocytes and macrophages, was completely downregulated in mDCs (*p* < 0.05), while the costimulatory ICOS-L was strongly increased in the iDCs (*p* < 0.01). Interestingly, the CCR7 marker, a chemokine receptor that confers the ability to DCs to migrate towards the lymph nodes, was upregulated both in iDCs and mDCs (*p* < 0.05 and *p* < 0.01, respectively), suggesting that Cabozantinib sustained the activation of DCs. The expression of the other DC markers such as HLA-DR, CD83, CD40, and CD86, was not altered after the addition of Cabozantinib.

To understand whether Cabozantinib modulated the endocytosis function of DCs, the uptake of the fluorescein isothiocyanate (FITC)-dextran by DCs (treated or untreated) was evaluated (**Figure 5C**).

Data were reported as the ratio between the MFI from positive (dextran uptake obtained after 1 h at 37°C) and negative (dextran uptake after 1 h at 4°C) samples. Results showed a trend (*p* = 0.08) in the reduction in the endocytic capacity of DCs treated with Cabozantinib compared to the not treated cells, suggesting a more mature status of the cells cultured in the presence of the drug.

## Discussion

The complex and heterogeneous network of dynamic interactions occurring among tumor cells, the surrounding microenvironment, and the immune cells offers a great opportunity for cancer therapeutic interventions. The introduction of immunotherapy as standard therapy in several tumor clinical setting enhances these opportunities (36). The finding that each therapeutic modality directly or indirectly may impact the antitumor immune response sustains the rational for designing novel sequence or combinatorial treatments.

Here, we describe *in vitro* that Cabozantinib induces ICD in prostate cancer cells and directly modulates DCs. Cabozantinib is a multitarget TKI and has proved its clinical efficacy in MTC, mRCC, and HCC. In prostate cancer, Cabozantinib did not appear to significantly increase overall survival (OS) (19), despite the promising early phase clinical trial results (37).

Cabozantinib exerts an important biological effect normalizing the tumor vasculature by the targeting of VEGF-R2. In prostate cancer mouse model, the therapeutic efficiency of Cabozantinib appears to be strongly associated with MET pathway, reducing cancer cell invasion, and this action synergizes with androgen receptor antagonist therapy (38). Both DU-145 and PC-3 cell lines expressed MET, and Cabozantinib treatment had a cytostatic effect, blocking cells in G1 phase, while no increase in apoptosis could be observed.

Interestingly, Cabozantinib induced an inhibition of the autophagic pathway in DU-145 cells, through an upregulation of the mTOR complex. This may be related to the high expression of the AXL that is observed in DU-145; this is also an RTK target for Cabozantinib. mTOR integrates extracellular molecule cues as such as nutrients and stress and triggers metabolic reprogramming of the cell, and its upregulation reduces the autophagic flux. AXL overexpression induces intensive cytoprotective autophagy that is involved in drug resistance (34). Therefore, interfering with AXL signaling appears to down-modulate the autophagic machinery in DU-145 cells. This effect was not observed in the PC-3 cells, in which mTOR is overexpressed at baseline (data not shown) and that shows poor expression of AXL. Interestingly, AXL inhibition was shown to concomitantly reduce autophagy and modulate ICD in a lung cancer cell model (34).

Our results show that the cellular stress induced by Cabozantinib resulted in the induction of ICD, in both cell lines, and this effect was more prominent in DU-145 cells.

ICD is a peculiar type of apoptosis that occurs following the exposure of cells to danger stimuli. It requires the activation of intracellular pathways leading to the simultaneous release of several DAMPs as HMGB1, ATP, and CRT exposure; each of them is sign of cellular distress and damage. DAMPs are recognized by professional antigen-presenting cells as such as DCs and determine DCs activation (39). So far, the induction of ICD by chemical therapeutics and radiations is a potential enhancer for the activation of the antitumor immune response by immunotherapy (29, 40). In a conditional prostate-specific Pten/Tp53 knockout mouse model, Cabozantinib treatment induced tumor clearance by massive infiltration of neutrophils, and this was dependent to CXCL12 and HMGB1 molecules that acted as chemoattractants (25).

Our results unequivocally demonstrated that Cabozantinib triggered ICD as defined by the simultaneous release of HMGB1 and ATP and the membrane exposure of CRT in human prostate cancer cells.

In other cancer cell lines, Cabozantinib failed to trigger ICD as in osteosarcoma cells (41). We also found that Cabozantinib treatment did not modulate HMGB1 release in renal cancer cell lines (**Supplementary Figure S2**). The different TKR expression levels and the complex transduction network that is activate downstream the receptors may make account for these distinct effects.

Extracellular vesicles (EVs) are intercellular conveyors of biological signals overcoming the need for cell-to-cell contact and transferring their biological cargo even to a distant acceptor cell, thus inducing its metabolic reprogramming (35). Tumor EVs may play a crucial role in DC activation and antigen presentation (42, 43). DAMPs can be cargo of EVs (44) and may contribute to disseminate the danger signals.

In our cell system, Cabozantinib induces the release of ICD markers (CRT and HMGB1) packed into the EVs and the HSP70 stress molecule. In addition, we observed that there was a flow of material between cancer cell lines and DCs. This release increased after the addition of Cabozantinib to tumor cells, suggesting that tumor cells were able to transfer antigens to DCs.

DCs are antigen-presenting cells (APCs) specialized to sense the danger in the tissues and activate and coordinate the innate and acquired immune responses, migrating to the lymph nodes. They are the main drivers of the antitumor immune response, operating as potent APCs, able to cross-present and to activate naïve T cells (45).

During differentiation, DCs express RTKs as such as VEGF-R2, and we have shown that Pazopanib, an anti-VEGFR TKI, modulated anticancer immunity and impacted DCs functions (10). Similarly, we found that Cabozantinib downregulated the monocyte markers CD14 and upregulated the migration receptor CCR7 and costimulatory marker ICOSL. These results were associated with a significant reduction in intracellular β-catenin. This pathway contribute to the balance between tolerance and immune response of DCs (46). β-Catenin expression is associated to DCs tolerance status, accompanied by the release of immunosuppressive molecules (47, 48), while β-catenin suppression or its absence led to activation of DCs (49). Accordingly, the endocytic ability of iDCs upon Cabozantinib treatment decreased: antigen uptake is a functional capacity that is downmodulated following DC maturation. Thus, Cabozantinib directly impacts DCs and may modulate their effector function.

Interestingly, Cabozantinib reduced immunosuppression in a castration-resistant prostate cancer mouse model. This effect was mainly due to reduced development and recruitment of myeloid-derived suppressor cells (MDSCs), leading to increased proliferation of intratumoral CD4^+^ and CD8^+^ T cells. This treatment, indeed, potentiated the response to ICI immunotherapy (24).

The results obtained in mouse models and the results herein provided strongly suggest that Cabozantinib may act as an immune modulator able to promote and sustain a more immune permissive microenvironment.

For prostate cancer, the improvement of the therapeutic schedules is a compelling need, in particular for those cancer patients who progress to the lethal stage of mCRPC (50).

The therapeutic combination of Cabozantinib with treatments that awaken patient immunity could provide benefit in patients with advanced cancer who usually do not respond to anti-PD-1/PD-L1 agents.

Indeed, the recent results of the COSMIC-021 phase 1b clinical trial (Cabozantinib in combination with Atezolizumab) are quite promising in the cohort of advanced prostatic cancer patients (23). In 101 high-risk mCRPC patients, the ORR was 27% (with 2% of complete response) with 88% of disease control rate. Considering the interesting results of COSMIC-021 trial, the phase III randomized, open-label CONTACT-02 clinical trial has been started. In this trial, Cabozantinib plus Atezolizumab is compared to the standard antihormone therapy (enzalutamide or abiraterone plus desametasone) in mCRPC patients (51).

It is therefore of great importance to characterize the off-target effects that Cabozantinib may exert on the immune system in order to optimize its use as a therapeutic agent in combination with ICIs immunotherapy in prostate cancer and in other tumor settings.

## Data Availability Statement

The original contributions presented in the study are included in the article/**Supplementary Material**. Further inquiries can be directed to the corresponding authors.

## Ethics Statement

The studies involving human participants were reviewed and approved by Comitato Etico del Policlinco Umberto I—Sapienza University, Rome, Italy (Rif.5282/08.04.2019). The patients/participants provided their written informed consent to participate in this study.

## Author Contributions

AR and MN designed the research and provided funding. FS and AP set up the methodologies. FS, AP, and AF performed experiments. IZ and HR contributed to the investigation, methodology, and validation. AR and CN supervised the study. FS, AR, and CN wrote the original draft. MN and IZ revised the manuscript. All authors contributed to the article and approved the submitted version.

## Funding

This research was funded by IPSEN and MIUR-Sapienza (RM120172B803DB14). The funder was not involved in the study design, collection, analysis, interpretation of data, the writing of this article, or the decision to submit it for publication.

## Conflict of Interest

The authors declare that the research was conducted in the absence of any commercial or financial relationships that could be construed as a potential conflict of interest.

## Publisher’s Note

All claims expressed in this article are solely those of the authors and do not necessarily represent those of their affiliated organizations, or those of the publisher, the editors and the reviewers. Any product that may be evaluated in this article, or claim that may be made by its manufacturer, is not guaranteed or endorsed by the publisher.
