# Supplementary material for: Immunogenic Cell Death and Immunomodulatory Effects of Cabozantinib
Source: Front Oncol. 2021 Oct 20;11:755433. doi: 10.3389/fonc.2021.755433 (PMC8564482; doi:10.3389/fonc.2021.755433)
Supplement: Supplementary file 1 [file DataSheet_1.pdf]

Supplementary Material and Methods

Renal cancer cell lines

Renal cancer RCC786-O was purchased from the American Type Culture Collection (ATCC; Washington, DC, NW) and cultured in RPMI (ATCC, 30-2001). Renal cancer RCC4plusVHL cells were purchased from the European Collection of Authenticated Cell Cultures (ECACC) and cultured in DMEM high glucose (Sigma-Aldrich), 2 mM Glutamine (Sigma-Aldrich) and G418 0.5mg/ml. Both cell lines were grown in the presence of 10% FCS.

Supplementary Table 1: Expression of TK targets by cancer cells

	**Glandular cells**	**DU-145**		**PC-3**		**monocyte-DC**		**CD14+ monocyte**	
	#ONLINE DATA BASE	#ONLINE DATA BASE	WB/FC	#ONLINE DATA BASE	WB/FC	##ONLINE DATA BASE	WB/FC	##ONLINE DATA BASE	WB/FC
**AXL**	144.6 pTPM	662 TPM	**+++**	165 TPM	**+**	NF	**-**	0.5 nx	**-**
**VEGFR-1**	1 pTPM	-	**-**	-	**-**	NF	**+**	0.3 nx<br>-	**+**
**VEGFR-2**	0.3 pTMP	-	**-**	2 TPM	**+/-**	NF	**+**	-<br>-	**-**
**FLT-3**	-	-	**-**	-	**-**	NF	**+**	2.1 nx	**+**
**C-MET**	163.7 pTPM	147 TPM	**+**	154 TPM	**+**	NF	**-**	0.4 nx<br>-	**+**
**C-KIT**	0.2 pTPM	1 TPM	**+/-**	-	**-**	NF	**+/-**	0.9 nx	**+/-**

NX= In the Human Protein Atlas, the NX value for every gene and tissue were calculated and visualized on the gene summary page together with the pTPM values for the individual samples. **Consensus** transcript expression levels for each gene were summarized in 74 human tissues based on transcriptomics data from three sources: HPA, GTEx and FANTOM5. The consensus normalized expression (NX) value for each gene and organ/tissue represents the maximum NX value in the three data sources.
pTPM= protein-transcripts per million
NF= not found on database
#https://www.ebi.ac.uk/gxa/home
##https://www.proteinatlas.org/

Supplementary Figure 1

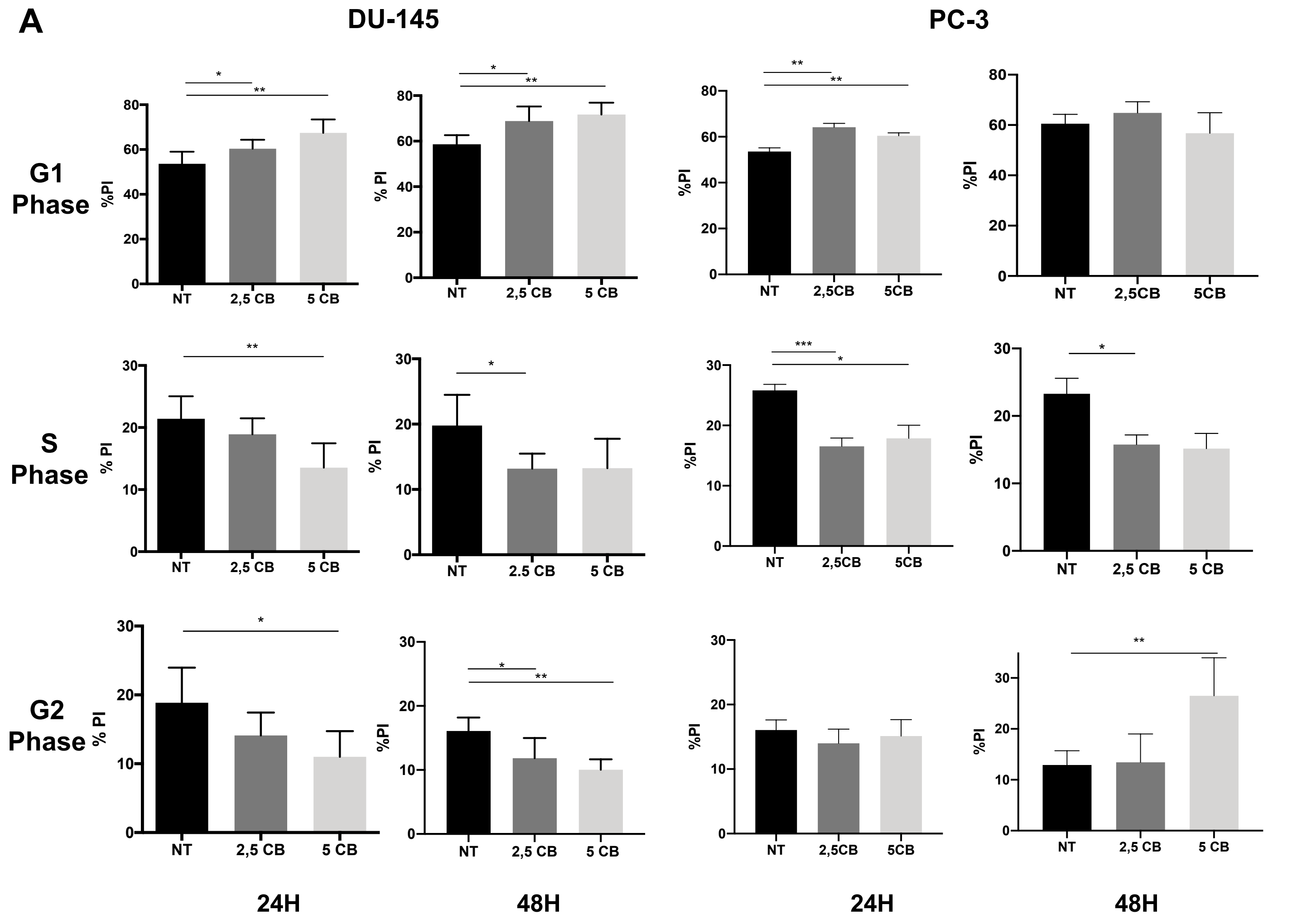

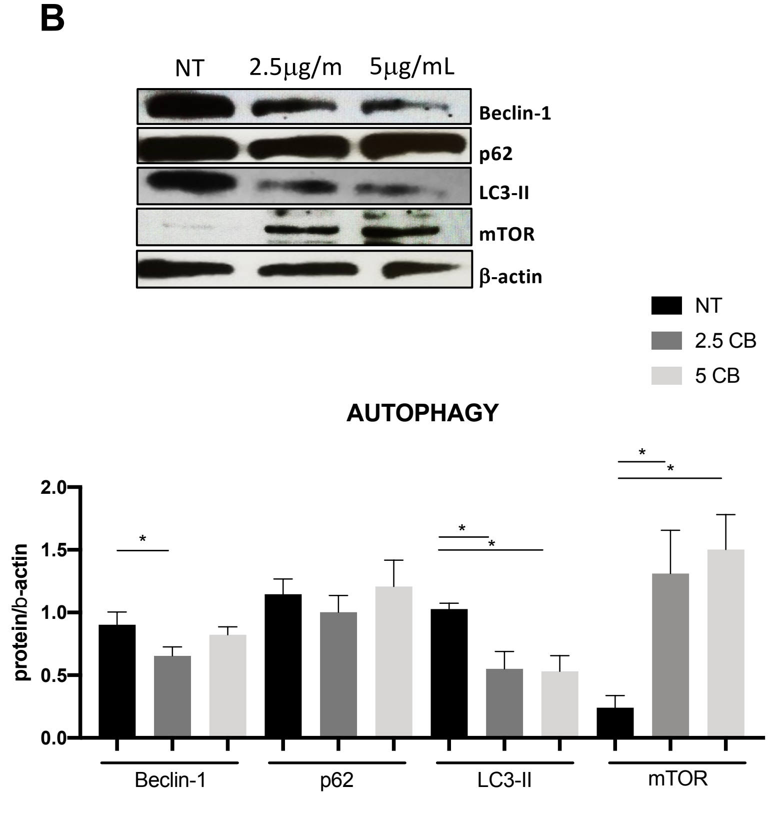

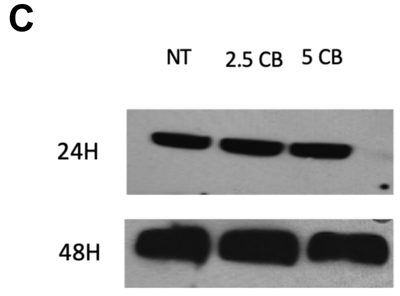

Supplementary Figure 1: DU-145 and PC-3 cells treated for 24 or 48 hours with different concentration of Cabozantinib (2.5 μg/mL and 5 μg/mL A) S and G2 phase of cancer cells ± Cabozantinib (2.5 μg/mL and 5 μg/mL) evaluated by low cytometry as % of Propidium Iodure (PI). The histograms represent the mean values±SEM of the percentage of G1 blocked cells of 3 separate experiments ± SEM from DU145 prostate cell line at 24 hours NT: not treated cells. *, *p* <0.05; **, *p*<0.01 of Student t-test. **B)** Western Blot analysis of autophagic markers in DU-145 cell extracts. Cells were untreated (NT), treated with Cabozantinib (2.5 μg/mL and 5 μg/mL). The histograms represent the mean±SEM of ratio of intensity lane of each analyzed protein and β-actin. **C)** HSP70 expression evaluated by Western Blot in DU-145 cells untreated or Cabozantinib treated (2.5 μg/mL and 5 μg/mL)

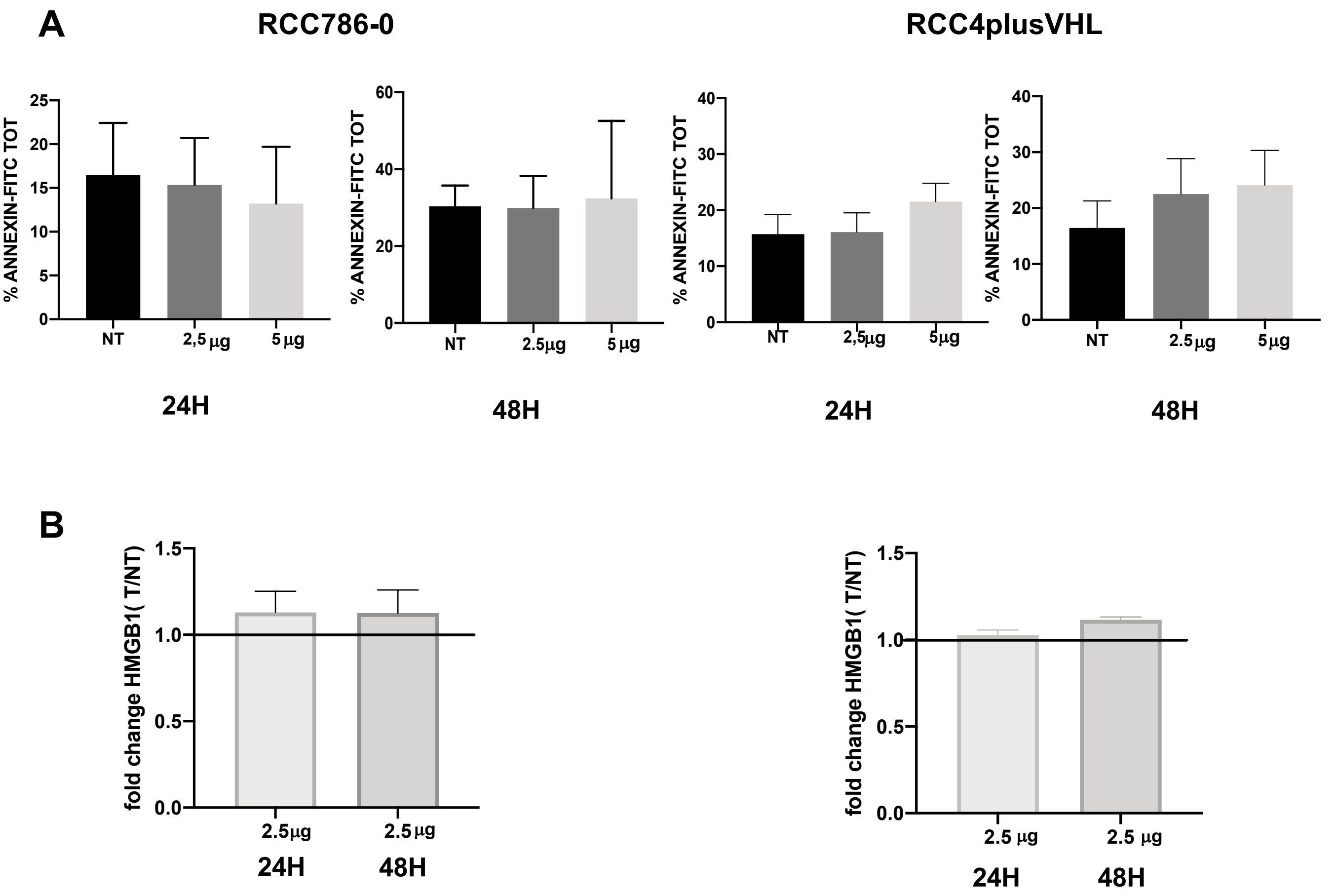

Supplementary Figure 2: RCC786-O and RCC4plusVHL cells treated with Cabozantinib: A) Apoptosis of cells treated for 24 or 48 h with Cabozantinib (2.5 µg/mL and 5 µg/mL). Histograms represent the mean values of percentage of apoptotic cells identified as Annexin positive of 3 independent experiments ± SEM. **B**) HMGB1 release upon Cabozantinib treatment. Histograms represent the ratio between the median values of HMGB1 released by treated cells (2.5 µg/mL) vs values of HMGB1 released by untreated cells (NT)
